# The Effects of Tight Lacing

**Published:** 1869-04

**Authors:** 


### The Effects of Tight Lacing.

The *London Lancet* thus sums up the evil effects of the fashionable custom of compressing the female thorax:

1. "Tight lacing seriously limits, almost annihilates the respiratory movements of the diaphragm; for the pinch comes just on that portion of the ribs to which the great muscle of inspiration is attached, and squeezes them together so as to throw it almost or altogether out of work.

2. The constant pressure of the corset on the muscles which should support the spine, gradually impairs their nutrition, so that they are no longer able to do their work, and the victim of tight-lacing feels wretched the moment her artificial supports are removed.

3. The hindrance to breathing with the diaphragm throws the work of respiration chiefly, if not altogether, upon the upper intercostal muscles and the muscles of the neck, and a permanent condition of imperfect aeration of the blood results, causing general languor and debility.

4. The abdominal viscera, especially the stomach and liver, are violently squeezed, and driven downward from their natural position; and the never failing result of this is impairment of digestion and assimilation. This dyspepsia may or may not be attended by pain and other obvious symptoms; these generally exist, but their absence does not imply the absence of mischief.

5. The uterine functions are always more or less perverted. Lastly, it may be mentioned that the pressure on the bust is very often productive of great suffering. The glands are so squeezed, and especially the nipples are so flattened, that when the time arrives for nursing an infant, the young mother finds either that she cannot suckle at all, or must do so in great misery, from inflamed nipples, abscess, &c.

Altogether, a more totally indefinable sin against the laws of health and good taste than tight lacing could not be found. If there be a spark of right and honest feeling left among our women, of whose purity of character we are so fond of boasting, now is the time for them to show it, by refusing to listen to the impudent sophistries like those to which we have referred.

